# Non-templated addition and template switching by Moloney murine leukemia virus (MMLV)-based reverse transcriptases co-occur and compete with each other

**DOI:** 10.1074/jbc.RA119.010676

**Published:** 2019-10-22

**Authors:** Madalee G. Wulf, Sean Maguire, Paul Humbert, Nan Dai, Yanxia Bei, Nicole M. Nichols, Ivan R. Corrêa, Shengxi Guan

**Affiliations:** New England Biolabs, Inc., Ipswich, Massachusetts 01938

**Keywords:** DNA polymerase, RNA, molecular biology, enzyme mechanism, reverse transcription, deep sequencing, non-templated addition, reverse transcriptase, single-cell RNA sequencing, template switching

## Abstract

Single-cell RNA-Seq (scRNA-Seq) has led to an unprecedented understanding of gene expression and regulation in individual cells. Many scRNA-Seq approaches rely upon the template switching property of Moloney murine leukemia virus (MMLV)-type reverse transcriptases. Template switching is believed to happen in a sequential process involving nontemplated addition of three protruding nucleotides (+CCC) to the 3′-end of the nascent cDNA, which can then anneal to the matching rGrGrG 3′-end of the template-switching oligo (TSO), allowing the reverse transcriptase (RT) to switch templates and continue copying the TSO sequence. In this study, we present a detailed analysis of template switching biases with respect to the RNA template, specifically of the role of the sequence and nature of its 5′-end (capped *versus* noncapped) in these biases. Our findings confirmed that the presence of a 5′-m^7^G cap enhances template switching efficiency. We also profiled the composition of the nontemplated addition in the absence of TSO and observed that the 5′-end of RNA template influences the terminal transferase activity of the RT. Furthermore, we found that designing new TSOs that pair with the most common nontemplated additions did little to improve template switching efficiency. Our results provide evidence suggesting that, in contrast to the current understanding of the template switching process, nontemplated addition and template switching are concurrent and competing processes.

## Introduction

Deep RNA-Seq has emerged as an important genomic tool as it provides a snapshot of the transcriptomes of tissues and cells under physiological and pathological conditions (1). Single-cell RNA-Seq (scRNA-Seq)^3^ has been recently used in the determination of cell lineage (2), for profiling the immune system (3), and in tumor evolution and metastasis (4). Due to the scarcity of RNA in a single cell, it is critical to avoid any material losses during the library preparation steps leading up to sequencing (5). In this context, template switching–based library preparation has emerged as a method of choice for scRNA-Seq because of its relatively simple workflow and low RNA input requirements (6). It has also been adapted to sequencing small RNAs as well as fragmented RNAs from liquid biopsies or microsomes (7). Furthermore, template switching has been used in long-read single-molecule approaches with third-generation sequencing platforms, including isoform (Iso)-Seq (PacBio SMART-Seq) (8) and nanopore RNA-Seq (Oxford Nanopore Technology) (9).

Template switching is an innate property of certain reverse transcriptases (RTs) to switch from one RNA template molecule to another (10–12). It is an essential mechanism of retroviral recombination and allows the synthesis of a complete cDNA copy (13–15). It was then applied to rapid amplification of cDNA ends (RACE), a technology designed to obtain full-length RNA (16). In template switching–based scRNA-Seq, a known adaptor sequence fused to an oligo(dT) serves as a primer to initiate the reverse transcription at the 3′-end of an mRNA. As the reverse transcription reaction progresses, the RT is able to switch from the 5′-end of the mRNA template to the 3′-end of an independent oligonucleotide with known sequence, called a template-switching oligo (TSO). As a result, the first-strand cDNA flanked by two known adaptors is synthesized in a single step. This cDNA will then serve as a template for PCR amplification and further library construction.

Despite its widespread use, the underlying mechanism by which template switching occurs remains elusive. The prevailing assumption is that MMLV-based reverse transcriptases add three nontemplated deoxycytidines (+CCC) to the 3′-end of the cDNA strand (17–19) via a critical terminal transferase activity. The protruding nontemplated cytidine nucleotides can then anneal to complementary guanosine nucleotides at the 3′-end of the TSO (rGrGrG-3′). Upon reaching the annealed intersection between the protruding cDNA and the incoming TSO, the RT continues synthesizing cDNA from the TSO template, thus effectively switching from one template to another. Similarly, studies have shown that WT HIV RT forms a complex with the primary RNA template, nascent DNA strand, and a single-stranded RNA donor template during strand-transfer events (36, 37). Further studies suggested that HIV RT can perform template switching (clamping) as long as the 3′-end of the primer donor strand and DNA/RNA template acceptor strands have complementary nucleotides (20, 21). The efficiency of template switching using HIV type 1 (HIV-1) RT seems to be affected by template–primer binding affinity and RNase H cleavage specificity (22). Other RT families also display terminal transferase activity. The avian myeloblastosis virus RT was found to add one or more nontemplated nucleotides (preferably +A) (23), whereas HIV-1 can preferentially add one to four nontemplated purine deoxynucleotides (+R to +RRRR) (24) with deoxyadenosine being the most common (25).

One technology that takes advantage of the terminal transferase activity of MMLV-based reverse transcriptases is CapSelect, which ligates a dsDNA adaptor to the cDNA, thus enriching full-length cDNAs for the analysis of mRNA sequences (26). As part of the development of CapSelect, Schmidt and Mueller (26) found that the terminal transferase activity of MMLV reverse transcriptases toward uncapped RNAs is not only less pronounced but also not cytidine-specific. In a more recent study, a comprehensive analysis was made to determine the optimal TSO 3′-end sequence to enhance template switching and understand base preferences during nontemplated nucleotide incorporation (27). Using randomized nucleotides on the 3′-end of the TSO (rN3rN2rN1–3′), Zajac *et al.* (27) demonstrated through sequencing experiments a strong preference for guanosine at the TSO N1 and N2 positions and suggested that the use of an rNrGrG-3′ motif may improve the efficiency of template switching. Although they reported that three to four deoxycytidines were primarily incorporated in the cDNA strand in a nontemplated fashion, there was no indication of any particular biases as for the RNA template sequence itself.

In light of the lack of in-depth experimental evidence on the origin of biases in template switching, we set to investigate how template switching is affected by both the RNA sequence and nature of its 5′-end (capped *versus* noncapped). We profiled the number and composition of the nontemplated nucleotides incorporated to the 3′-end of the cDNA strand and demonstrated that the makeup of the RNA template 5′-end plays a key role in swaying the RT terminal transferase activity. We confirmed that the presence of a 5′-m^7^G cap enhances the efficiency of template switching and that the cap itself can act as a template for the reverse transcriptase. Furthermore, we provide evidence that template switching and nontemplated addition are concurrent and competing processes. Our results give a new perspective on the template switching process and underscore the contribution of the RNA template sequence as a source of bias during library preparation.

## Results

### The 5′-end of RNA affects the template switching efficiency

**Figure 1.**
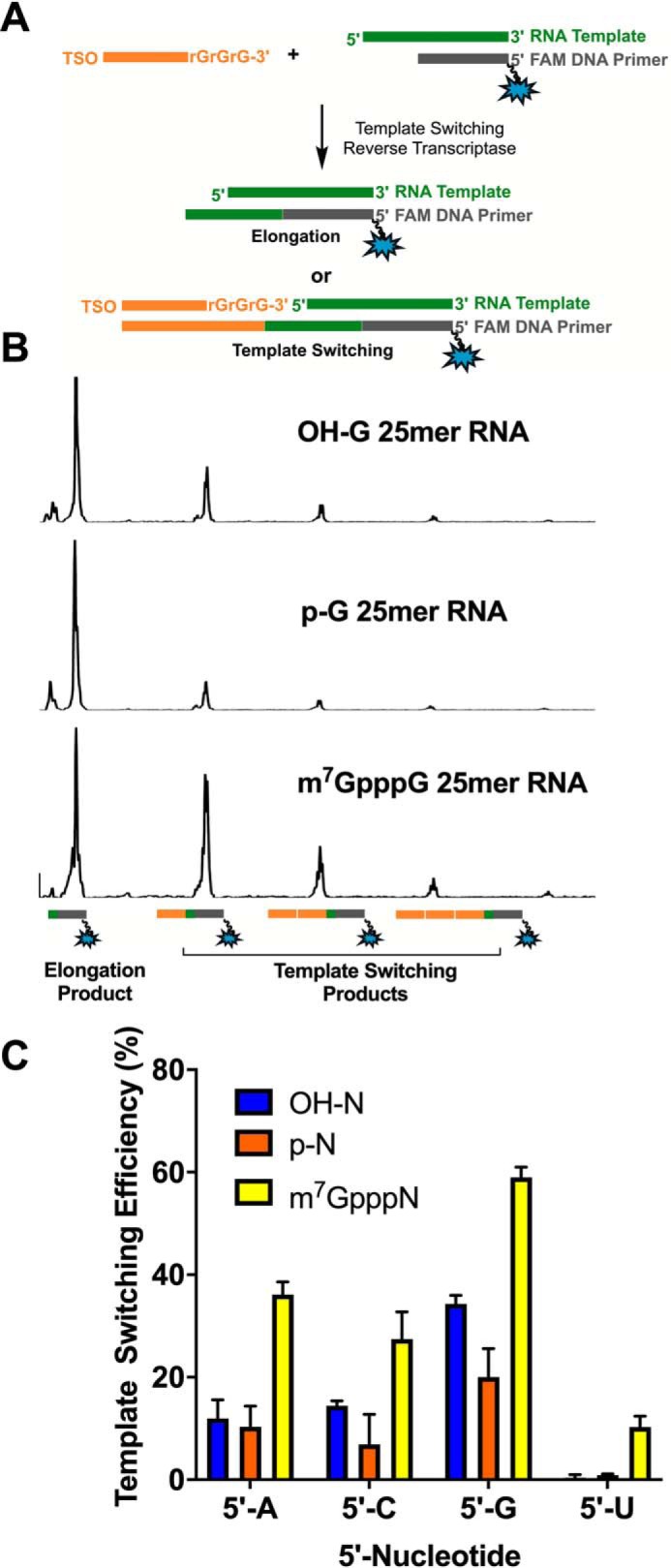
**Template switching efficiency is affected by both the first nucleotide and 5′ modification of the RNA template.** *A*, schematic illustration of the assay to determine template switching efficiency through CE. *B*, representative trace of CE data for G-25mer-RNA with different 5′ modifications (5′-OH, 5′-*p*, or 5′-m^7^G cap). *C*, quantification of template switching efficiency. Data represent mean ± S.D. (*error bars*) of *n* = 6 independent experiments.

To determine whether the 5′-end of RNA affects template switching efficiency, we designed a set of 25mer RNA oligonucleotides, varying the identity of the first nucleotide (A, C, G, or U) and the modification at the 5′-end (5′-hydroxyl (5′-OH), 5′-monophosphate (5′-*p*), or 5′-m^7^G cap). By utilizing a defined RNA sequence, we minimized the dependence of template switching on internal sequence variation. We carried out the template-switching reverse transcription by using a 5′-FAM–labeled DNA primer and a TSO that contained three terminal guanosine ribonucleotides at the 3′-end (rGrGrG-3′ TSO) (Fig. 1*A*). As it has been reported that MMLV-based reverse transcriptases add multiple nontemplated cytidines to the 3′-end of the cDNA strand during reverse transcription (17), the rGrGrG-3′ TSO is thought to provide the highest template switching efficiency. Capillary electrophoresis (CE) was used to quantify the primer elongation and template switching products, including concatemers, that form when multiple template switching events occurred on the same cDNA (Fig. 1*B*).

Template switching was significantly affected by the first nucleotide of the RNA template (Fig. 1*C*). For RNA oligonucleotides with the same 5′-end modification, template switching was most efficient when the first nucleotide was G followed by C or A. RNA oligonucleotides starting with U had the lowest template switching efficiency. Template switching was also affected by the modification of the 5′-end of the RNA. For RNAs with the same first nucleotide, m^7^G-capped oligonucleotides showed significantly greater template switching efficiency than 5′-*p* or 5′-OH oligonucleotides. In addition, we examined the template switching bias on m^7^G-capped and 5′-*p* uncapped RNA oligonucleotides with six MMLV-based reverse transcriptases, including MMLV RT, Template Switching RT, SuperScript II, SuperScript IV, Maxima H Minus, and SMARTScribe in both their manufacturers' suggested buffers (Fig. S1, *A* and *B*) and in a commonly used template switching buffer (Smart-seq2 buffer) (28) (Fig. S1, *C* and *D*). Although the overall template switching efficiencies varied significantly when the RTs were used in their own buffers, similar efficiencies were observed when the reverse transcription reactions were performed in the Smart-seq2 buffer (Fig. S1, *A* and *B*). More importantly, all the RTs investigated in this study showed the same pattern of template switching products with respect to the RNA template sequence, with RNAs starting with G being the most effective and those with U being the least effective. Additionally, capped RNA showed higher template switching efficiencies than uncapped RNA regardless of the RT.

### The template switching bias is marginally affected by the internal RNA sequence

**Figure 2.**
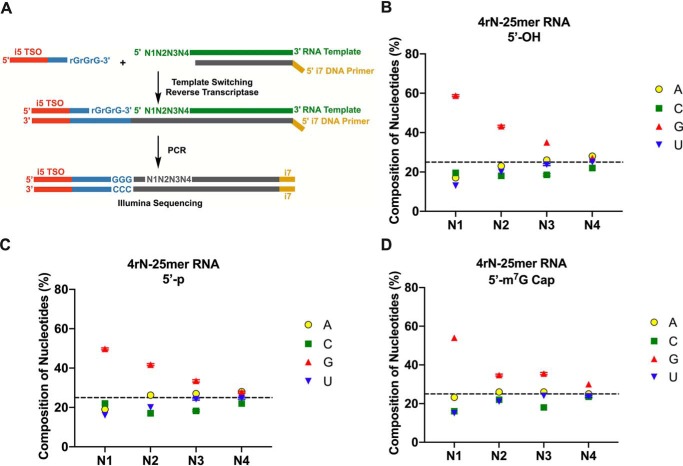
**Template switching bias decreases for nucleotide positions more distal from the 5′-end.** *A*, schematic illustration of the library preparation method to determine the template switching bias for RNA templates with the first four nucleotides randomized. *B*, *C*, and *D*, averaged composition of nucleotides at each of the first four positions (N1, N2, N3, and N4) for RNA templates with 5′-OH (*B*), 5′-*p* (*C*), or 5′-m^7^G cap (*D*). Data represent mean ± S.D. (*error bars*) of *n* = 4 independent experiments.

To further analyze the effect of the internal RNA sequence on the bias of template switching, we synthesized RNA oligonucleotides with the first four nucleotides randomized. Again, the 5′-end of the RNA oligonucleotide was either a 5′-OH, 5′-*p*, or 5′-m^7^G cap (Fig. 2*A*). The relative composition of the first four ribonucleotides (N1:N2:N3:N4) in the RNA template was determined by deep sequencing to account for any nucleotide misrepresentation introduced during RNA synthesis (Fig. S2*A*) (29). We found that the relative distribution of the four ribonucleotides was consistent at each position with a slight overrepresentation of pyrimidines in all synthetic oligonucleotides (Fig. S2, *B–D*). We then performed the template-switching reverse transcription on these RNA oligonucleotides and constructed Illumina libraries to determine the extent of biases (Fig. 2, *B–D*). The sequencing data from template switching experiments were normalized to the relative composition of the first four ribonucleotides in the respective synthetic templates. The results suggested that regardless of whether the 5′-modification was capped or uncapped, there was a strong preference for guanosine at position N1 (Fig. 2, *B–D*), which is consistent with the template switching efficiency results described in Fig. 1*C*. Uncapped RNA templates also favored guanosine at position N2. The nucleotide preference decreased along each of the remaining randomized nucleotides with N4 showing minimal contribution to the overall bias.

### Profiling the nontemplated nucleotide addition reveals diverse terminal transferase activity

**Figure 3.**
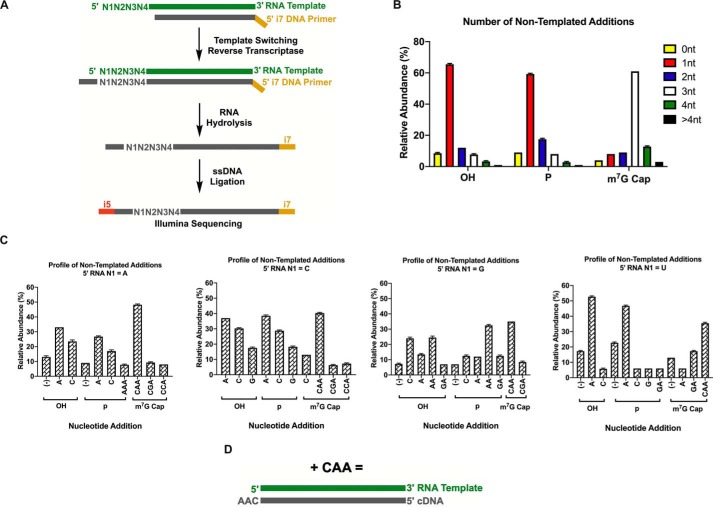
**Nontemplated addition profile is predicated on the identity of the first nucleotide and 5′ modification of the RNA template.** *A*, schematic illustration of the assay to determine the profile of nontemplated addition through deep sequencing. *B*, number of nontemplated nucleotides (*nt*) added across RNA template with 5′-OH, 5′-*p*, or 5′-m^7^G cap. *C*, nontemplated nucleotide addition profile categorized according to the first nucleotide of RNA template. Only nucleotide additions with more than 5% relative abundance are shown. *D*, schematic representation of +CAA nontemplated addition product formed during reverse transcription. Data represent mean ± S.D. (*error bars*) of *n* = 4 independent experiments. Data shown in *B* and *C* are from deep sequencing analysis. Data from MS analysis are shown in Fig. S3.

The RT terminal transferase activity is critical for the process of template switching. In an attempt to explain the origin of the biases observed for templates starting with a guanosine, we profiled the number and identity of the nontemplated nucleotides added by the RT to the 3′-end of the cDNA across different RNA template 5′-ends. Two independent methods, deep sequencing and MS, were used to determine the nontemplated addition profile (Figs. 3*A* and S3*A*). It is important to note that these experiments were carried out in the absence of a TSO.

For deep sequencing, a library of RNA templates with four randomized nucleotides at the 5′-end was created using ssDNA ligation (Fig. 3*A*). We found that the number of nontemplated nucleotides varied significantly depending on the 5′-end RNA modification (Fig. 3*B*). Whereas m^7^G-capped templates led to cDNA products with the addition of three nontemplated nucleotides as the major species, the 5′-OH and 5′-*p* RNAs gave rise to products with primarily one nontemplated nucleotide. These results confirm earlier evidence that an MMLV-based reverse transcriptase will add a different number of nontemplated nucleotides depending on whether the RNA is capped or not (26). Next, we investigated the identity of these nontemplated nucleotide additions (Fig. 3*C*). Surprisingly, unlike what has been reported previously (17, 27), m^7^G-capped RNAs gave rise to +CAA as the major nontemplated nucleotide addition regardless of the RNA nucleotide at position N1 (for simplicity and as shown in Fig. 3*D*, +CAA represents the sequence of nontemplated deoxynucleotides added to the 3′-end of cDNA as follows: first a dC followed next by a dA and then another dA consecutively). In general terms, a higher terminal transferase activity evidenced by an increase in nontemplated additions correlated with a higher template switching efficiency (Figs. 3*C* and 1*C*).

Interestingly, the first nontemplated nucleotide incorporated across all m^7^G-capped RNAs was almost always dC, pointing toward a contribution of the guanosine cap on “templating” the first addition, which has been suggested before (18). However, we did not observe any +CCC addition regardless of the 5′-end modification. This was particularly intriguing as the majority of the template switching protocols utilized a purportedly matching rGrGrG-3′ TSO. As the ssDNA ligation step used for deep sequencing library preparation could have introduced additional biases, we analyzed the cDNA products of Fig. S3*A* by intact MS to confirm our results. Although MS does not directly inform on the nucleotide order of the nontemplated addition, it enables an unbiased assessment of the number and composition of nucleotides added. Based on the patterns observed for the sequencing data, we could infer from the MS results that the most prevalent species observed for capped RNAs was indeed +CAA and to a lesser extent +CCA and +CC (Fig. S3*B*). No significant difference in the nontemplated addition profile was observed by the varying nucleotides at the N1 position of the RNA template with the exception of N1 = U, which favored +A and rarely added more than +2 nucleotides. This apparent discrepancy between sequencing and MS results for N1 = U may be attributed to the fact that the MS analysis is semiquantitative and was carried out with a single template (N1 = U, N2 = U, N3 = A, and N4 = G), whereas the deep sequencing results were averaged from a randomized pool comprising all potential nucleotide combinations at each of the three positions N2, N3, and N4.

To further confirm that the observed nontemplated addition profile generally applies to other MMLV-based reverse transcriptases, we repeated the reverse transcription reaction of one m^7^G-capped RNA (N1 = G) with two additional enzymes, MMLV and SuperScript II reverse transcriptases, both in their manufacturer's recommended buffer and in the Smart-seq2 buffer. We found that the +CAA product was the major species formed with either enzyme in the Smart-seq2 buffer (Fig. S4, *B* and *D*), which is consistent with the results obtained with Template Switching RT. In line with the observed low template switching efficiencies (Fig. S1*A*), the reverse transcription reactions performed in the manufacturer's recommended buffers resulted in cDNA products containing only one nontemplated addition, favoring the formation of the +C species (Fig. S4, *A* and *C*).

For uncapped RNA templates, there was much less discrimination against the incoming nucleotide during the nontemplated nucleotide addition. With both 5′-OH and 5′-*p* templates, the most abundant cDNA products contained +A, +C, and +G additions except in the case of RNAs with N1 = U where +A was observed predominantly (Figs. 3*C* and S3*B*). In uncapped templates where N1 = G, besides +A and +C, two-nucleotide additions, +AA and +GA, were also observed, corroborating the hypothesis that the ability of MMLV-based reverse transcriptase to template switch is directly associated with its terminal transferase activity. It is also noteworthy that 5′-OH and 5′-*p* RNAs led to a considerable amount of cDNA products with no nontemplated nucleotides added (denoted by “−”). The general preference for a single nontemplated addition across all uncapped RNAs was also confirmed by MS analysis (Fig. S3*B*).

### Alternative TSO designs do not improve template switching efficiency

**Figure 4.**
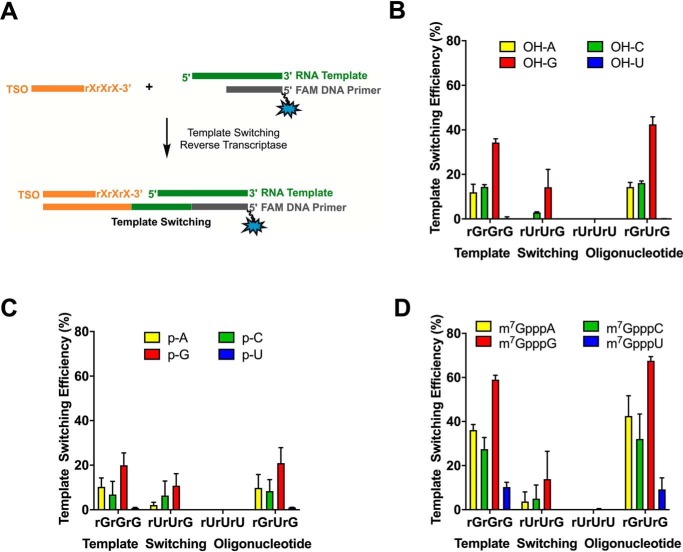
**Comparing the performance of different TSOs on template switching.** *A*, schematic illustration of the assay to determine template switching efficiency through capillary electrophoresis. *B–D*, template switching efficiencies for rGrGrG-3′ TSO, rUrUrG-3′ TSO, rUrUrU-3′ TSO, and rGrUrG-3′ TSO in a reverse transcription reaction with RNA templates differing by the identity of the first nucleotide and the nature of the 5′-end modification: 5′-OH (*B*), 5′-*p* (*C*), and 5′-m^7^G (*D*). Data represent mean ± S.D. (*error bars*) of *n* = 6 independent experiments.

As it has been reported that template switching relies on the successful annealing between the TSO 3′-end and the cDNA 5′ protruding nontemplated nucleotides (10), we designed a series of TSOs to match the most common nontemplated additions detected in our experiments. For example, rUrUrG-3′ TSO was designed to capture the abundant +CAA addition found with most m^7^G-capped RNAs. An rGrUrG-3′ TSO was designed as a variant of the rGrGrG-3′ TSO to account for the additional +CA motif observed in the nontemplated addition, and an rUrUrU-3′ TSO was designed to account for the prevalent +A addition, particularly for 5′-OH and 5′-*p* RNA templates. All template switching experiments with the new TSOs were compared with the standard rGrGrG-3′ TSO (Fig. 4*A*).

Surprisingly, although +CAA was the most common nontemplated addition observed with m^7^G-capped RNAs in the absence of a TSO, the matching rUrUrG-3′ TSO showed minimal template switching and was markedly inefficient compared with the rGrGrG-3′ TSO (Fig. 4*D*). The poor performance of rUrUrG-3′ TSO suggests that the +CAA addition may not play a significant role during the template switching process. Interestingly, the rGrUrG-3′ TSO provided template switching efficiencies comparable to the rGrGrG-3′ TSO. The fact that, in the absence of a TSO, +CAC/+CCC were noticeably rare additions and that the highest template switching efficiencies were observed for rGrUrG-3′ and rGrGrG-3′ TSOs led us to consider that a TSO-mediated extension of the nascent cDNA seemed to be competing with an outright TSO-independent terminal transferase process.

Similar to what was observed for capped RNAs, the template switching efficiencies for uncapped RNAs (5′-OH and 5′-*p*) with the newly designed TSOs were also largely inferior to those obtained with the rGrGrG-3′ TSO. Only the rGrUrG-3′ TSO showed efficiencies comparable to the rGrGrG-3′ TSO. Interestingly, the rUrUrU-3′ TSO did not trigger any appreciable template switching even though uncapped RNAs (in particular when N1 was uridine) showed significant amounts of +A incorporation by the RT terminal transferase activity alone (Fig. 4, *B* and *C*). We speculate that the abundant +A nontemplated addition in 5′-U RNAs would result in weaker hydrogen bonding relative to a C–G base pair and thus lead to weaker TSO/nontemplated addition interactions and less efficient template switching.

### Extra nucleotides are added in the cDNA product during the transient junction of a capped RNA template and TSO

**Figure 5.**
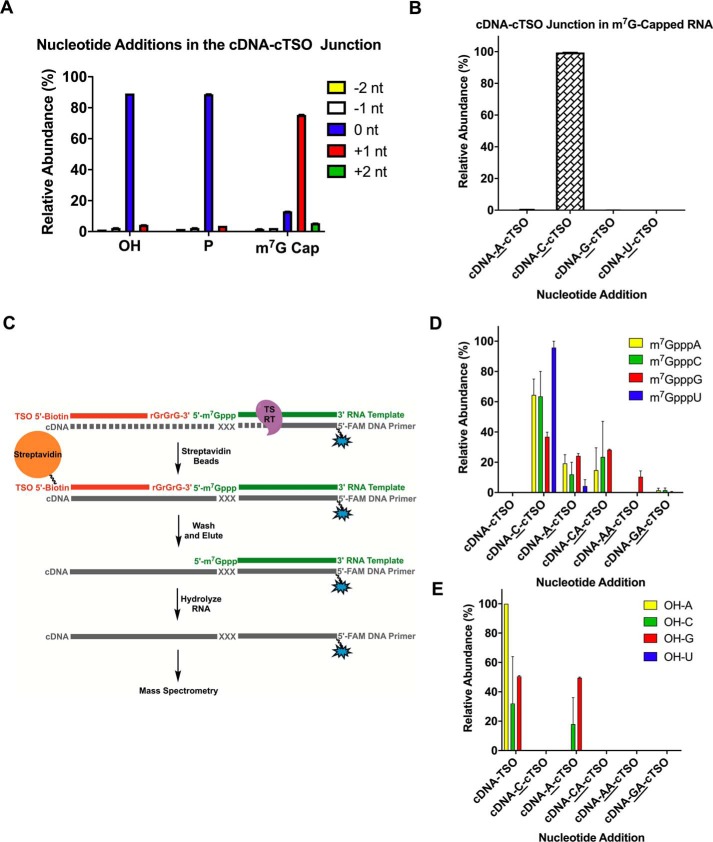
**Nucleotide profile at the cDNA–cTSO junction.** *A*, number of extra nucleotides (*nt*) added at the cDNA–cTSO junction determined by deep sequencing. *B*, identity of the extra nucleotide addition at the cDNA–cTSO junction for m^7^G-capped RNA templates. Sequencing data represent mean ± S.D. of *n* = 4 independent experiments. *C*, schematic illustration of the assay to determine the nucleotide profile at the cDNA–cTSO junction through MS. *D*, nucleotide addition profile at the cDNA–cTSO junction for m^7^G-capped RNA templates determined by MS. *E*, nucleotide addition profile at the cDNA–cTSO junction for 5′-OH RNA templates determined by MS. Mass spectrometry data represent mean ± S.E. (*error bars*) of *n* = 2 independent experiments. *TS*, template switching.

Given the conflicting results observed between the nontemplated addition profile in the absence of a TSO and the template switching efficiencies in the presence of corresponding TSOs, we decided to investigate the nucleotide sequence in the cDNA product at the interface between the RNA template and TSO strands (for clarity, the reverse transcription product comprising the complements of these two strands is represented by cDNA–cTSO). The sequencing results from the randomized library in Fig. 2*A* were utilized to determine the nucleotide composition at the cDNA–cTSO junction. Our data indicated that template-switched products from m^7^G-capped RNAs had predominately one extra nucleotide in the cDNA–cTSO junction (Fig. 5*A*), and the identity of this nucleotide was almost always a deoxycytidine (Fig. 5*B*). In contrast, uncapped RNAs overwhelmingly (∼90% of the reads) led to no extra nucleotides in the junction (Fig. 5*A*).

To independently verify the sequencing profile at the cDNA–cTSO junction, we carried out a slightly different experiment using MS. Because mass accuracy decreases with the increase in size of the cDNA product, we designed a TSO with a 5′-biotin modifier to limit concatemer formation (30) and at the same time allow enrichment of the template-switched products. After a template-switching reverse transcription reaction, the cDNA product was captured using streptavidin beads, eluted, and treated with sodium hydroxide to hydrolyze the RNA template (Fig. 5*C*). Similar to what was observed by deep sequencing, MS analyses indicated an extra +C present at the cDNA–cTSO junction with all m^7^G-capped RNAs (Fig. 5*D*). We also detected minor incorporations of +A, +CA, and +AA; however, we cannot rule out by MS alone whether some of these additions (particularly of +A) may have occurred at the 3′-end of the cDNA–cTSO strand (across the biotin group) and not at the cDNA–cTSO junction. When uncapped RNAs were subjected to the same template switching conditions, no nontemplated +C addition was observed at the cDNA–cTSO junction (Fig. 5*E*).

The major addition of a single +C at the cDNA–cTSO junction conveys the idea that the m^7^G cap structure itself may act as a template for the RT during cDNA synthesis, making the first nontemplated addition of +C, in reality, templated. It is unclear at this point whether this extra cytidine may be responsible for seeding the hybridization with the incoming rGrGrG-3′ TSO. The lack of other nucleotides at the cDNA–cTSO junction suggests that template switching may immediately occur as soon as the first +C is added (Fig. 5*B*). Hence, once the template switching starts, the nucleotide incorporation at the 3′-end of cDNA strand is dictated by the sequence of the TSO. Although this does not necessarily explain why other TSOs do not give rise to efficient template switching, we can only presume that the stability of the TSO/cDNA interaction may be a key factor in this process.

### Nontemplated nucleotide additions and template switching are concurrent processes

**Figure 6.**
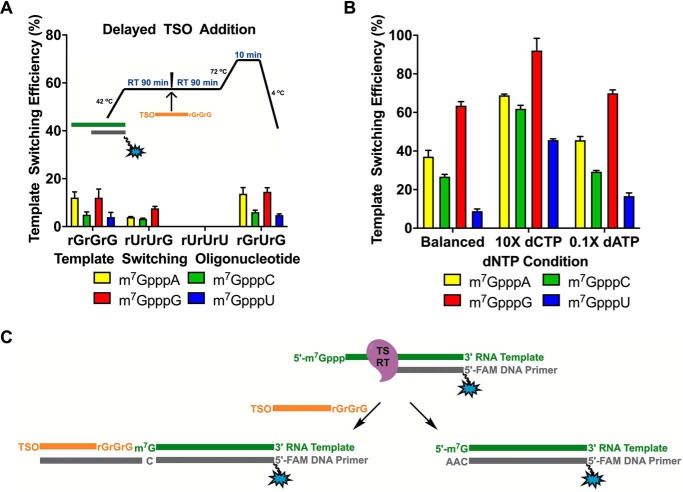
**Nontemplated addition and template switching are concurrent processes.** *A*, template switching efficiency with delayed TSO addition. The *arrow* indicates the time point at which the TSO was added during the reverse transcription reaction. *B*, template switching efficiency with equimolar (balanced) and nonequimolar dNTP mixtures (10× dCTP or 0.1× dATP). *C*, proposed relationship between template switching and nontemplated addition. Data represent mean ± S.D. (*error bars*) of *n* = 6 independent experiments. *TS*, template switching.

To further study the relationship between nontemplated addition and template switching, we performed the template switching reaction with a delayed addition of TSO with the goal of permitting TSO-independent nontemplated addition to take place prior to the strand switching process (Fig. 6*A*). Not surprisingly, under these conditions, the template switching efficiency was reduced by 2–5-fold (Figs. 6*A* and 4*A*). The results indicate that the process of template switching happens concurrently with that of nontemplated addition. If the two processes happened sequentially and independently, the template switching efficiencies may have remained the same regardless of the timing of the TSO addition.

Based on the premise that a +C addition favors template switching whereas a +A disfavors it, we sought to enhance the overall efficiency of the switching process by altering the availability of dCTP and dATP during the reverse transcription reaction. Concordantly, when we utilized 10-fold excess dCTP (10× dCTP), the template switching efficiency increased by nearly 2-fold for all capped RNAs (Fig. 6*B*). Even for the 5′-m^7^GpppU RNA, which otherwise exhibited poor template switching, there was a dramatic increase in efficiency, bringing it to a level comparable with the other capped RNAs. As expected, nontemplated addition in the absence of a TSO and with 10× dCTP resulted in a marked increase in deoxycytidine incorporation (Fig. S5). Interestingly, these were the only conditions under which the +CCC extension alone was observed with any abundance. These results align with our hypothesis that the nontemplated deoxycytidines likely enhance template switching by creating a stronger, more stable interaction with the TSO.

Next, we tested the reverse transcription reaction with an unbalanced dNTP mixture containing 110 of dATP (0.1× dATP). We reasoned that if the amount of nontemplated deoxyadenosines could be suppressed, more nontemplated cytidines could be added by the RT on average, which in turn would increase the overall template switching efficiency. On that account, the 0.1× dATP mixture produced a smaller but distinguishable increase in the template switching efficiency across all capped RNAs (Fig. 6*B*).

Taking into consideration the cumulative results of this study, some new light can be shed on the mechanism of template switching by MMLV-based reverse transcriptases (Fig. 6*C*). In the presence of a capped RNA template and a DNA primer, a cDNA strand is generated to the end of the RNA template. As soon as the first nontemplated deoxycytidine is added, template switching can occur. If further nontemplated additions take place, most commonly of deoxyadenosines, template switching is suppressed. Therefore, we propose that nontemplated nucleotide addition and template switching happen concurrently, not sequentially, in the context of the reverse transcription reaction.

## Discussion

Uncovering and understanding inherent biases of template switching–based methods is critical for RNA-Seq at the single-cell level. In view of the well-documented biases found in traditional ligation approaches for the introduction of 5′-sequencing adaptors (31–33), many efforts to rationally improve current template-switching reverse transcription protocols, either through the design of novel TSOs or modifying the reaction conditions, are underway in both industrial and academic settings. The first important lesson we learned in this study is that, for uncapped RNAs, there is a marked bias depending upon the identity of the template 5′-nucleotide. A clear preference for guanosine was noticeable in the first nucleotide at the 5′-end. These results raise concerns for the application of template switching–based RNA-Seq workflows with uncapped RNAs, such as small RNAs and highly degraded RNAs, including RNAs extracted from formalin-fixed paraffin-embedded tissue samples. The use of template switching for library preparation from uncapped RNAs will likely cause certain transcripts, such as those with a 5′-terminal guanosine, to be overrepresented in the sequencing reads. Biases are still present with m^7^G-capped RNAs; however, considering that mRNA transcription start sites usually comprise G or A and rarely U, the overall bias effect for single-cell RNA-Seq is not as drastic as with uncapped RNAs. Fundamentally, understanding the mechanisms by which RTs template switch is imperative to minimize RNA-Seq data distortion, particularly in low-input sequencing.

It has been generally accepted that three to four nontemplated 3′-terminal deoxycytidines are added to the nascent cDNA strand during reverse transcription (18, 26). Through a combination of sequencing and MS techniques, we found that, in the absence of a TSO, capped RNAs generally favor the incorporation of three nontemplated nucleotides, of which +CAA was the most abundant under our conditions. Uncapped RNAs led to the incorporation of fewer nucleotides (mainly +1) and no strong preference for cytidine; in fact, +A, +C, and +G additions were all detected in significant levels. This deviation from the reported +CCC nucleotide extension is further supported by two recent studies that looked at the polymerase activity of MMLV reverse transcriptases on DNA substrates. The enzyme was able to add not only 3′-C tails to the DNA but also A-, G-, or T-tails, and certain reaction additives could enhance or suppress particular tailing events (34, 35). Therefore, it seems more plausible that MMLV-based reverse transcriptases have much more fluidity in their terminal transferase activity than was accepted previously. Therefore, modifying reaction conditions is likely to influence the nature and extent of the nontemplated addition, as was demonstrated in this study.

What is perhaps more interesting is how these nontemplated additions relate to the process of template switching. Zajac *et al.* (27) analyzed the nontemplated addition in template switching products by randomizing the 3′-end of the TSO and used that knowledge to infer the nontemplated incorporation preference. This approach reports only on the nontemplated addition that were favorably enriched in the sequencing libraries as a result of the template switching process. Furthermore, their interpretation of the data assumed a direct correlation between the nucleotides in the TSO and the identity of otherwise nontemplated additions, which our study shows is not always congruent. Although their readout does not necessarily reflect the whole picture behind nontemplated addition, many of their findings agree with the results reported here: for example, the strong preference for guanosine at the 3′-position of the TSO and for the incorporation of deoxycytidine as the first nontemplated nucleotide in the cDNA strand. Besides deoxycytidine, the authors indicated that other bases can be incorporated by the RT, which is consistent with the array of nontemplated addition observed in our study. Zajac *et al.* (27) also alluded to the possibility of redesigning the TSO as a way to target cDNA strands featuring low-frequency nontemplated bases. However, as we found here, due to the concurrent nature of template switching and TSO-independent nontemplated addition, the use of a partially randomized TSO, such as rNrGrG-3′, is unlikely to improve the capture of potentially missing cDNA strands.

Cumbie *et al.* (18), in their approach for detection of transcription start sites, identified a “cap signature” of an unencoded guanosine in the second-strand cDNA, which was attributed to the fact that the 5′-m^7^G cap may be acting as a template for a cytidine in the first-strand cDNA synthesis. The templating properties of the m^7^G cap would certainly be supported by the results of our study, especially when noting the strong preference for +C as the first “nontemplated” nucleotide of capped *versus* uncapped RNA templates. However, Cumbie *et al.* (18) still recognize the sequential three- to four-nontemplated cytidine extension as being the accepted mechanism of MMLV-based reverse transcriptases. Therefore, our results provide the first direct evidence of template switching and nontemplated addition acting concurrently. Our results further bring into question whether annealing between the rGrGrG-3′ TSO and nontemplated addition is even necessary for template switching to occur. It is conceivable that instead the RT has some preference for binding a particular TSO, making template switching favorable, or further that the cap structure itself somehow stalls the RT, allowing more time for the TSO to interact and allow template switching to occur. Additional studies are certainly needed to address these questions.

Because of its simplicity, template switching–based RNA-Seq is increasingly gaining popularity, particularly for single-cell and low-input RNA library preparation. Given that both 5′- and 3′-sequencing adaptors can be incorporated into the cDNA strand in a single reverse transcription reaction, template switching is an attractive choice for minimal RNA input. A potential drawback to single-cell RNA-Seq is the fact that the efficiency of template switching is generally low (5, 38); however, due to advances in instrumentation and library preparation protocols, sensitivity no longer appears to be of critical concern (6, 28). Our study provides important insights into the template switching process and paves the way for further improvements in both efficiency and specificity. With a better understanding of the process by which RTs add nontemplated nucleotides and template switch, there continues to be real promise for a truly unbiased RNA-Seq platform, which will ultimately enable the analysis of the transcriptome with even more detail and accuracy.

## Experimental procedures

### Materials

All reagents were from New England Biolabs (NEB), Ipswich, MA, unless otherwise stated. 5′-OH and 5′-*p* 25mer RNAs, TSOs, and primers were synthesized by Integrated DNA Technologies (Coralville, IA). The RNA template 4rN-25mer-RNA-FAM was synthesized using the “hand-mix” option with an equimolar ratio of all four bases (Integrated DNA Technologies). 5′-Triphosphate RNAs were synthesized as described previously (39). RNA templates, primers, and TSO sequences are described in Table S1.

### Preparation of capped RNA templates (m^7^GpppN-25mer)

Capping of 5′-triphosphate 25mer RNAs (5 nmol; 5′-*ppp*NUAGAACUUCGUCGAGUACGCUCAA-3′) was performed at a 500-μl reaction volume using the Vaccinia Capping System. Briefly, 10 μm 5′-triphosphate RNA, 1× Capping Buffer (50 mm Tris-HCl, 5 mm KCl, 1 mm MgCl_2_, and 1 mm DTT, pH 8, at 25 °C), 30 μm GTP, 200 μm SAM, 5 units of pyrophosphatase (catalog number M2403), and 500 units of Vaccinia Capping Enzyme (catalog number M2080) were incubated overnight at 37 °C. Capped RNAs were purified by phenol/chloroform extraction using Phase Lock Gel tubes (5Prime, Hilden, Germany; catalog number 2302810) followed by PAGE. Purified capped RNAs were resuspended in 50 μl of water, and the final oligonucleotide concentration was estimated with a NanoDrop spectrophotometer (Thermo Fisher Scientific, Waltham, MA).

### Reverse transcription reaction assays for CE and MS analysis

Six MMLV-based reverse transcriptases were evaluated for their ability to template switch: SuperScript II (Thermo Fisher Scientific, catalog number 18064014), SuperScript IV (Thermo Fisher Scientific, catalog number 18090050), Maxima H Minus (Thermo Fisher Scientific, catalog number EP0753), SMARTScribe (Clontech, catalog number 639538), M-MuLV reverse transcriptase (New England Biolabs, catalog number M0253; MMLV RT), and Template Switching RT (New England Biolabs, catalog number M0466; the Template Switching RT enzyme mixture contains a murine RNase inhibitor; to be consistent with the other reverse transcription assays, here we utilized the RT alone). For the reverse transcription reactions, a 20mer 5′-FAM V5 primer was designed to be complementary to the 3′-end of the 25mer RNA templates (5′-OH, 5′-*p*, or 5′-m^7^G–capped RNAs). The TSOs used in this study comprised a common 39-nucleotide DNA sequence at the 5′-end and a variable three-nucleotide RNA sequence at the 3′-end. TSO and primer sequences can be found in Table S1. Unless otherwise noted, Template Switching RT, an MMLV-based reverse transcriptase with reduced RNase H activity (40), was used throughout this study.

A nontemplated-addition reverse transcription reaction (no TSO present; 30-μl total volume) contained 1 μm RNA template (30 nmol), 1× Template Switching RT Buffer (catalog number B0466), 2 mm dNTP solution mixture (catalog number N0447), 0.5 μm 5′-FAM V5 primer, and 400 units of Template Switching RT. The reaction was performed at 42 °C for 90 min followed by a 10-min heat-denaturation step at 72 °C. The template RNA was hydrolyzed by adding 1 m sodium hydroxide (10 μl) and 0.5 m EDTA, pH 8.0 (10 μl), to the reaction and heating to 65 °C for 15 min. The cDNA was purified using the Oligo Clean & Concentrator (Zymo Research, Irvine, CA; catalog number D4061) and analyzed by MS.

A template-switching reverse transcription reaction (10-μl total volume) contained 0.1 μm RNA template, 1× Template Switching RT Buffer (catalog number B0467), 1 mm dNTP solution mixture, 30 nm 5′-FAM V5 primer, 1 μm TSO, and 100 units of Template Switching RT. The reaction was performed at 42 °C for 90 min followed by a 10-min heat-denaturation step at 72 °C. The reverse transcription reaction was directly analyzed by capillary electrophoresis without purification.

For template-switching reverse transcription reactions using the biotin-rGrGrG-3′ TSO, hydrophilic streptavidin magnetic beads (catalog number S1421) were used for purification prior to RNA hydrolysis. Briefly, the reverse transcription reaction was incubated with the beads for 10 min, then washed (500 mm NaCl, 20 mm Tris-HCl, pH 7.5, and 1 mm EDTA), rinsed with a low-salt buffer (150 mm NaCl, 20 mm Tris-HCl, pH 7.5, and 1 mm EDTA), and eluted with a solution of 10 mm Tris-HCl, pH 7.5, and 1 mm EDTA. The eluted RNA:cDNA was then subjected to RNA hydrolysis as described above and purified using the Oligo Clean & Concentrator. The purified cDNA was analyzed by MS.

### Capillary electrophoresis

In brief, 1 μl of a 1–20 nm sample was added to 10 μl of a mixture of HiDi^TM^ formamide and GeneScan^TM^-120 LIZ^TM^ Size Standard (Thermo Fisher Scientific, catalog number 4324287) and analyzed in an Applied Biosystems (Foster City, CA) 3130xl (16-capillary array) or 3730xl (96-capillary array) Genetic Analyzer with a 36-cm-long capillary coated with POP7 polymer. Data were collected via Applied Biosystems Data Collection software and processed with Applied Biosystems Peak Scanner software (41).

### Mass spectrometry

Intact oligonucleotide MS analysis was performed at Novatia LLC (Newtown, PA) using on-line desalting, flow-injection electrospray ionization on a Thermo Fisher Scientific LTQ-XL ion trap mass spectrometer and analyzed with ProMass Deconvolution software. The composition of the nontemplated nucleotide addition to cDNA was determined by comparison with calculated molecular masses.

### Library preparation to profile the four randomized nucleotides of synthetic RNA template

The 4rN-25mer-RNA-FAM oligonucleotide was 5′-phosphorylated with T4 polynucleotide kinase and then further converted to the m^7^G-capped derivative by treatment with guanosine 5′-pyrophosphorylimidazolide in a Mn(II)-promoted reaction (42) followed by guanine-*N*7 methylation using Vaccinia Capping Enzyme and SAM as the methyl donor. A standard reverse transcription reaction (10-μl total volume) contained 2 μm RNA template (5′-OH, 5′-*p*, or 5′-m^7^G–capped 4rN-25mer-RNA-FAM), 1× ProtoScript II Reaction Buffer, 1 mm dNTP solution mixture, 0.5 μm i7 primer (see supporting information for primer sequence), 10 mm DTT, and 200 units of ProtoScript II (catalog number M0368). The reaction was performed at 25 °C for 5 min and 42 °C for 30 min. The RNA template was hydrolyzed by adding 2 μl of 1 m NaOH and incubated at 65 °C for 15 min. The cDNA was purified with a Monarch PCR & DNA Cleanup kit (catalog number T1030) using the oligonucleotide cleanup protocol. A poly(G) tail was added to the 3′-end of cDNA (50-μl reaction in 1× Terminal Transferase Reaction Buffer) using 10 units of Terminal Transferase (catalog number M0315) and 2 mm dGTP. The reaction was incubated at 37 °C for 1 h. After the dG tailing, a pair of primers (Primer-universal-15C and one NEBNext Index primer for Illumina) was used to amplify the cDNA. After bead purification using NEBNext Sample Purification Beads (catalog number E7767), PCR products were mixed with 40% PhiX and subjected to Illumina iSeq-100 sequencing (2X45).

### Library preparation to determine bias of template-switching reverse transcription

A template-switching reverse transcription reaction (10-μl total volume) contained 0.1 μm RNA template (5′-OH, 5′-*p*, or 5′-m^7^G–capped 4rN-25mer-RNA-FAM), 1× Template Switching RT Buffer, 1 mm dNTP solution mixture, 30 nm i7 primer, 1 μm biotin-i5 rGrGrG-3′ TSO (see supporting information for TSO sequence), and 100 units of Template Switching RT. The reaction was performed at 42 °C for 90 min followed by a 10-min heat-denaturation step at 72 °C. Then 1 μl of the reverse transcription reaction was subjected to PCR amplification with NEBNext Universal and Index primers (catalog number E7335) using NEBNext High-Fidelity 2× PCR Master Mix (catalog number M0541). The libraries were cleaned up with NEBNext Sample Purification Beads (catalog number E7767) and sequenced on an Illumina iSeq-100 as described above.

### Library preparation to profile cDNA nontemplated nucleotide addition

A nontemplated-addition reverse transcription reaction (no TSO present; 50-μl total volume) contained 0.1 μm RNA template (5′-OH, 5′-*p*, or 5′-m^7^G–capped 4rN-25mer-RNA-FAM), 1× Template Switching RT Buffer, 1 mm dNTP solution mixture, 30 nm i7 primer, and 500 units of Template Switching RT. The reaction was performed at 42 °C for 90 min followed by a 10-min heat-denaturation step at 72 °C. After the reaction, 2.5 units of RNase H (catalog number M0297) and 25 units of RNase I_f_ (catalog number M0243) were added to the reaction and incubated at 37 °C for 20 min to hydrolyze the RNA template. The cDNA was purified using an Oligo Clean & Concentrator. The purified cDNA was incubated in a 20-μl reaction with 750 nm 5′-A*pp*DNA (see supporting information for sequence), 1× NEBuffer 1, 5 mm MnCl_2_, and 2 μm Thermostable 5′-A*pp*DNA/RNA Ligase (catalog number M0319) at 65 °C for 60 min to perform 3′ single-strand cDNA ligation. The reaction was inactivated at 90 °C for 3 min. The ligated products were subjected to PCR amplification with NEBNext Universal and Index primers (catalog number E7335) using NEBNext High-Fidelity 2× PCR Master Mix (catalog number M0541). The libraries were cleaned up with NEBNext Sample Purification Beads (catalog number E7767) and sequenced on an Illumina iSeq-100 as described above.

### Sequencing data analysis

Custom Python scripts were written to extract Illumina reads with the constant region of the template RNA. For profiling the composition of the four randomized nucleotides in the RNA template and the biases in the template-switching reverse transcription reaction, the four variable positions were extracted and analyzed. For profiling of the nontemplated-addition reverse transcription reaction, the sequences between the constant region of the RNA template and single-stranded A*pp*-DNA adaptor were extracted and sorted according to the first nucleotide of RNA template. All sequencing FASTQ files are available as supporting information. Graphical representation and statistics were performed using GraphPad Prism 8.

## Author contributions

M. G. W., S. M., P. H., and N. D. data curation; M. G. W. formal analysis; M. G. W., I. R. C., and S. G. writing-original draft; Y. B. and N. M. N. resources; I. R. C. and S. G. supervision; I. R. C. and S. G. writing-review and editing.

## Supplementary Material

Supporting Information

## Footnotes

3 The abbreviations used are:

scRNA-Seq: single-cell RNA-Seq
RT: reverse transcriptase
TSO: template-switching oligo
MMLV: Moloney murine leukemia virus
HIV-1: HIV type 1
FAM: fluorescein
CE: capillary electrophoresis
ssDNA: single-stranded DNA.
