# Supplementary material for: Non-templated addition and template switching by Moloney murine leukemia virus (MMLV)-based reverse transcriptases co-occur and compete with each other
Source: J Biol Chem. 2019 Oct 22;294(48):18220–31. doi: 10.1074/jbc.RA119.010676 (PMC6885630; doi:10.1074/jbc.RA119.010676)
Supplement: Supporting Information [file supp_294_48_18220__index.html]

Non-templated addition and template switching by Moloney murine leukemia virus (MMLV)-based reverse transcriptases co-occur and compete with each other — MMLV-type reverse transcriptase template switching — Supporting Information 

# Non-templated addition and template switching by Moloney murine leukemia virus (MMLV)-based reverse transcriptases co-occur and compete with each other

## Supporting Information

- Supporting Information (to be published online) - Sequencing Data
- Supporting Information (to be published online) - Supporting Table and Figures
