# Supplementary material for: Non-templated addition and template switching by Moloney murine leukemia virus (MMLV)-based reverse transcriptases co-occur and compete with each other
Source: J Biol Chem. 2019 Oct 22;294(48):18220–31. doi: 10.1074/jbc.RA119.010676 (PMC6885630; doi:10.1074/jbc.RA119.010676)
Supplement: Supporting Information [file supp_RA119.010676_155253_1_supp_413298_pz3tx7.docx]

**Supporting Information**

**Table 1**: **Oligonucleotide templates, reverse transcription primers, and TSO sequences** (unless otherwise specified RNA nucleotides are preceding by “r”, DNA nucleotides are preceding by “d”; “N” represents a 25:25:25:25 mixture of canonical nucleotides).

| **RNA Templates** | **Sequence** |
| --- | --- |
| 4rN-25mer-RNA-FAM | 5’-(N)(N)(N)(N)AACUUCGUCGAGUACGCUCAA-FAM-3’, |
| A-25mer-RNA | 5’-AUAGAACUUCGUCGAGUACGCUCAA-3’ |
| C-25mer-RNA | 5’-CUAGAACUUCGUCGAGUACGCUCAA-3’ |
| G-25mer-RNA | 5’-GUAGAACUUCGUCGAGUACGCUCAA-3’ |
| U-25mer-RNA | 5’-UUAGAACUUCGUCGAGUACGCUCAA-3’ |
| **RT DNA Primer** | **Sequence** |
| 5’-FAM V5 primer | 5’-FAM-d(TTGAGCGTACTCGACGAAGT)-3’ |
| i7 Primer | 5’-d(GACTGGAGTTCAGACGTGTGCTCTTCCGATCT(N)(N)(N)(N)(N)TTGAGCGTACTCGACGAAG)-3’ |
| **TSO** | **Sequence** |
| rGrGrG-3’ TSO | 5’-d(GCTAATCATTGCAAGCAGTGGTATCAACGCAGAGTACAT)rGrGrG-3’ |
| rUrUrG-3’ TSO | 5’-d(GCTAATCATTGCAAGCAGTGGTATCAACGCAGAGTACAT)rUrUrG-3’ |
| rUrUrU-3’ TSO | 5’-d(GCTAATCATTGCAAGCAGTGGTATCAACGCAGAGTACAT)rUrUrU-3’ |
| rGrUrG-3’ TSO | 5’-d(GCTAATCATTGCAAGCAGTGGTATCAACGCAGAGTACAT)rGrUrG-3’ |
| Biotin-rGrGrG-3’ TSO | 5’-Biotin-d(AAGCAGTGGTATCAACGCAGAGTACAT)rGrGrG-3’ |
| Biotin-i5-rGrGrG-3’ TSO | 5’-d(TACACTCTTTCCCTACACGACGCTCTTCCGATCT)rGrGrG-3’ |
| **Amplification Primer** | **Sequence** |
| Primer-universal-15C | 5’-d(AATGATACGGCGACCACCGAGATCTACACTCTTTCCCTACACGACGCTCTTCCGATCTCCCCCCC CCCCCCCCD)-3’ |
| **Adaptor** | **Sequence** |
| 5’-A*pp*DNA | 5’-rA*pp-*d(AGATCGGAAGAGCGTCGTGTAGGGAAAGAGTGT)-3SpC3-3’ |

**Figure S1. Relative template switching efficiency patterns across different MMLV-type reverse transcriptases.** Determination of template switching efficiency for six different reverse transcriptases (Template Switching RT, SuperScript II, SuperScript IV, Maxima H Minus, SMARTScribe, and MMLV RT) in their manufacturer’s recommended buffer for capped **(A)** and uncapped p-25mer **(B)** RNA. Determination of template switching efficiency of the same six reverse transcriptases in the Smart-seq2 buffer for capped **(C)** and uncapped p-25mer **(D)** RNA. Data represent mean ± SD (error bars) of n=3 independent experiments.

** Figure S2. Composition of the first four nucleotides of synthetic 4rN-25mer-RNA-FAM template.** (**A**) Schematic illustration of library preparation method to determine the composition of the first four randomized nucleotides in the synthetic 4rN-2mer-RNA-FAM template. Composition of the first four randomized nucleotides in the RNA template with a 5’-OH (**B**), 5’-*p* (**C**), or 5’-m^7^G cap (**D**). Data represent mean ± SD (error bars) of n=4 independent experiments.

**Figure S3.** **Mass spectrometry analysis of non-templated nucleotides added by Template Switching RT.** (**A**) Schematic illustration of assay to determine the profile of non-templated addition through mass spectrometry. (**B**) Nucleotide profile of the most abundant non-templated additions categorized according to the first nucleotide (N1) and the nature of the 5’ end modification of the RNA template (5’-OH, 5’-*p*, or 5’-m^7^G). A cut-off of 5% relative abundance was used for graphing individual non-templated additions for each RNA template. For comparative purposes, the profiles of +CAA and of no non-templated nucleotides added (-) were included for each capped RNA template, regardless of abundance. Data represent mean ± SD (error bars) for n=2 independent experiments.

**Figure S4. Mass spectrometry analysis of non-templated nucleotides added by wild-type MMLV and SuperScript II reverse transcriptases.** Nucleotide profile of all non-templated additions for the m^7^G-capped (N1 = G) RNA template using MMLV RT in either the manufacturer’s recommended buffer (**A**) or in Smart-seq2 buffer (**B**). Nucleotide profile of all non-templated additions for the m^7^G-capped (N1 = G) RNA template using SuperScript II RT in either the manufacturer’s recommended buffer (**C**) or in Smart-seq2 buffer (**D**). Data represent mean ± SD (error bars) of n=2 independent experiments.

**Figure S5. Comparison of the profile of non-templated nucleotide addition between equimolar and non-equimolar dNTP conditions through mass spectrometry.** The use of an excess of dCTP (10x dCTP) leads to an increase in deoxycytidine and a decrease in deoxyadenosine incorporation at the cDNA strand across m^7^G-capped RNA templates. The RNA template sequence is represented by identity of the nucleotide N at position 1: (**A**) N1 = A, (**B**) N1 = C, (**C**) N1 = G, and (**D**) N1 = U.
